# Longitudinal Assessment of Quality of Life Following Molecular Testing for Indeterminate Thyroid Nodules

**DOI:** 10.1245/s10434-021-10375-6

**Published:** 2021-07-22

**Authors:** Max A. Schumm, Dalena T. Nguyen, Jiyoon Kim, Chi-Hong Tseng, Amy Y. Chow, Na Shen, Masha J. Livhits

**Affiliations:** 1 Section of Endocrine Surgery, Department of Surgery, University of California Los Angeles David Geffen School of Medicine, 10833 Le Conte Ave. 72-227 CHS, Los Angeles, CA 90095 USA; 2 Department of Biostatistics, UCLA Fielding School of Public Health, Los Angeles, CA USA; 3 Division of General Internal Medicine and Health Services Research, Department of Medicine, University of California Los Angeles David Geffen School of Medicine, Los Angeles, CA USA; 4 Division of Endocrinology, Diabetes, and Metabolism, Department of Medicine, University of California, Los Angeles David Geffen School of Medicine, Los Angeles, CA USA

## Abstract

**Background:**

Molecular testing can refine the risk of malignancy in cytologically indeterminate thyroid nodules and can reduce the need for diagnostic thyroidectomy. However, quality of life (QOL) in patients mananged with molecular testing is not well studied.

**Objective:**

We aimed to assess the QOL of patients undergoing surveillance after a benign molecular test result, or thyroidectomy after a suspicious molecular test result.

**Methods:**

This prospective longitudinal follow-up of the Effectiveness of Molecular Testing Techniques for Diagnosis of Indeterminate Thyroid Nodules randomized trial utilized the Thyroid-Related Patient-Reported Outcome, 39-item version (ThyPro-39) to assess the QOL of patients with indeterminate cytology on thyroid fine needle aspiration (FNA) biopsy. All patients underwent molecular testing at the time of initial FNA. A mixed-effect model was used to determine changes in QOL over time.

**Results:**

Of 252 eligible patients, 174 completed the assessment (69% response rate). Molecular test results included 72% (*n =* 124) benign and 28% (*n =* 50) suspicious. ThyPro-39 scores of benign molecular test patients were unchanged from baseline (following initial FNA and molecular test results) to 18 months of ultrasound surveillance. Baseline symptoms of goiter, anxiety, and depression were more severe for patients with suspicious compared with benign molecular test results. At a median of 8 months after thyroidectomy, suspicious molecular test patients reported improved symptoms of goiter, anxiety, and depression.

**Conclusion:**

A benign molecular test provides sustained QOL throughout ultrasound surveillance, without worsening anxiety or depression relating to the risk of malignancy. Definitive surgery results in improvement of QOL in patients with suspicious molecular tests.

The use of medical imaging has made thyroid nodules increasingly prevalent in clinical settings.^1^ Fine needle aspiration (FNA) biopsy is the standard diagnostic modality to evaluate for malignancy; however, 10–30% of nodules fall into an indeterminate category (Bethesda class III or IV), with a 10–40% risk of malignancy.^2^–^4^ In the US alone, approximately 100,000 cytologically indeterminate thyroid nodules are evaluated annually.^5^

Historically, indeterminate nodules were managed with repeat FNA or thyroid lobectomy for definitive diagnosis; however, the advent of molecular testing has allowed for greater diagnostic insights and de-escalation of surgical care.^6^ Approximately 40% of all indeterminate thyroid nodules undergo molecular marker testing in the US.^7^ Results of molecular testing often guide subsequent treatment, with ultrasound surveillance generally recommended for nodules with benign molecular test results and surgery recommended for nodules with suspicious test results.

Thyroid cancer has a disproportionately negative effect on quality of life (QOL) compared with malignancies with a worse prognosis.^8^–^11^ In addition, discrepancies exist between physician-estimated QOL in thyroid cancer survivors and QOL reported by patients.^12^ This impact on QOL may extend to the growing population of patients with indeterminate thyroid nodules who are managed with molecular testing. Data on QOL of patients with cytologically indeterminate nodules are very limited. Patients with indeterminate cytology who have benign molecular test results report similar QOL scores compared with patients with benign cytology, suggesting that a benign molecular test provides adequate reassurance that the risk of malignancy is low.^13^ However, no prior studies have assessed the QOL of patients undergoing surveillance after a benign molecular test result or in those undergoing thyroidectomy after a suspicious molecular test. These are important measures to study given the increasing number of patients with cytologically indeterminate nodules who are managed with molecular testing and long-term surveillance.

In this longitudinal study, we aimed to measure the QOL of patients with indeterminate thyroid nodules who had benign molecular test results through the course of ultrasound surveillance. We hypothesized that the initial reassurance provided by a benign molecular test diagnosis would be sustained over time. We also compared the pre- and postoperative QOL of patients with indeterminate thyroid nodules and suspicious molecular test results who underwent thyroidectomy.

## Materials and Methods

### Study Population

All patients aged 18 years and older who underwent FNA biopsy with indeterminate cytology results throughout the University of California, Los Angeles (UCLA) Health System from August 2017 to January 2020 were eligible for enrollment. Those excluded were patients under the age of 18 years, those with concurrent thyroid malignancy, those who received nondiagnostic molecular test results, and non-English-speaking patients, due to the inability to provide our electronic QOL assessment in a language other than English. This study was approved by the UCLA Institutional Review Board.

### Study Design

Biopsies were performed within UCLA Health by the patient’s endocrinologist, endocrine surgeon, or radiologist. At the time of the FNA, an additional sample was routinely collected for potential molecular testing and reflexively sent for all patients with indeterminate cytopathology (Bethesda 3: atypia of undetermined significance or follicular lesion of undetermined significance; or Bethesda 4: follicular neoplasm or suspicious for follicular neoplasm).^2^ At the time of FNA, all patients were counseled that if their results were indeterminate they would undergo molecular testing. Patients with indeterminate nodules were block-randomized by month to either Afirma Genomic Sequence Classifier (GSC) or ThyroSeq v3 molecular tests in protocol with a concurrent randomized controlled trial (RCT) performed by our group.^14^ The performance characteristics of both GSC and Thyroseq v3 have been confirmed in independent validation studies.^15^,^16^ The results of molecular testing guided subsequent treatment, with ultrasound surveillance generally recommended for nodules with benign molecular test results and surgical resection recommended for nodules with suspicious test results. All operations were performed using an open/conventional approach. Patients were counseled that benign test results conferred a <5% risk of malignancy, while suspicious results conferred an approximately 50% risk of malignancy, depending on the specific test results.^15^,^16^ Patients managed nonoperatively were followed with ultrasound surveillance every 6–12 months. The results from ultrasound surveillance are being prospectively evaluated and will be published elsewhere.

Variables extracted from the medical record included patient age, sex, thyroid-stimulating hormone (TSH) level, coexisting hypothyroidism (defined as TSH >4.7 mU/L), largest nodule diameter reported by cervical ultrasonography, Bethesda diagnostic category, molecular test performed and result, operation details (including procedure type [thyroid lobectomy vs. total thyroidectomy] and surgical pathology), and observation duration (time zero was defined as day of FNA). No data were collected on subject’s race or ethnicity.

### Quality-of-Life (QOL) Assessment

Molecular test results typically took 2–3 weeks to return after FNA and were communicated to the patient by the treating physician within 1 week (3–4 weeks after FNA; by electronic medical record messaging or phone call). The primary treating physician for most patients was their endocrinologist (approximately 10 in total, all with expertise in treating thyroid nodules). The endocrinologist was generally the initial physician who notified the patient of their cytology and molecular test results, for both suspicious and benign cases. Following communication of the molecular test result and plan of treatment, patients were contacted by designated research personnel, either by email or telephone, to enroll in the study and complete electronic QOL assessments administered every 6 months from the time of molecular test diagnosis. Reminder emails and phone calls for incomplete QOL surveys were conducted up to five times at each time point. Survey responses were categorized into three groups based on survey completion time from FNA: baseline (0–4 months after FNA), early (4–12 months after FNA), and late (12–24 months after FNA). Survey responses were grouped into two molecular test result categories—benign or suspicious. As no significant differences in QOL were found between the two molecular tests, the QOL scale scores for GSC and Thyroseq v3 were combined for analysis.

QOL assessment was continued after surgical intervention, when applicable. Patients with suspicious molecular test results who completed QOL surveys at baseline and at least 1 month after thyroidectomy were included, while those who underwent thyroidectomy prior to completing a baseline survey or underwent thyroidectomy with benign molecular tests were excluded from analysis.

### QOL Survey Instrument

This study utilized the Thyroid-Related Patient-Reported Outcome, 39-item version (Thy-Pro-39) to capture a patient’s QOL. Thy-Pro-39 is a validated disease-specific questionnaire with 11 scales (goiter symptoms, hyperthyroid symptoms, eye symptoms, tiredness, cognitive complaints, anxiety, depression, emotional susceptibility, impaired social life, impaired daily life, and appearance) and one single QoL composite score.^17^ The categories of the ThyPro-39 that were relevant to thyroid nodules and potential thyroid cancer were analyzed, including symptoms of goiter, anxiety, depression, emotional susceptibility, impaired social life, impaired daily life and appearance. Experience of each symptom related to these categories was answered by patients given the following choices, with the number of points allocated in parentheses: not at all (0), a little (1), some (2), quite a bit (3), and very much (4). A QOL scale score between 0 and 100 was then calculated for each category, such that a higher score represents a worse QOL.^17^

### Statistical Analysis

Descriptive data were generated from demographic and clinical information to characterize the study populations based on age, sex, nodule size, TSH level, Bethesda category, molecular test and result, and time to survey completion. An unstructured mixed-effect model was used for repeated measurement data to determine significant differences in QOL scores while accounting for the correlation between repeated measurement of each patient. This was performed for benign molecular test result patients at baseline, and early and late follow-up, and in suspicious molecular test patients at baseline, early follow-up, and postoperatively following thyroidectomy. Our model used all longitudinal data and adjusted for a fixed effect of time to survey completion (baseline, early, and late follow-up) and molecular test result and a random component to account for correlations between repeated measurements within subjects. By using all available data and accommodating unbalanced or incomplete data, this analysis was more efficient than direct comparisons. Univariate logistic regression analyses were used to assess ThyPro-39 scores of benign molecular test patients from time of baseline survey completion to 18 months of follow-up to create a model of QOL development over time. All *p*-values were two-sided, and a *p*-value <0.05 was considered statistically significant.

## Results

**Fig. 1.**
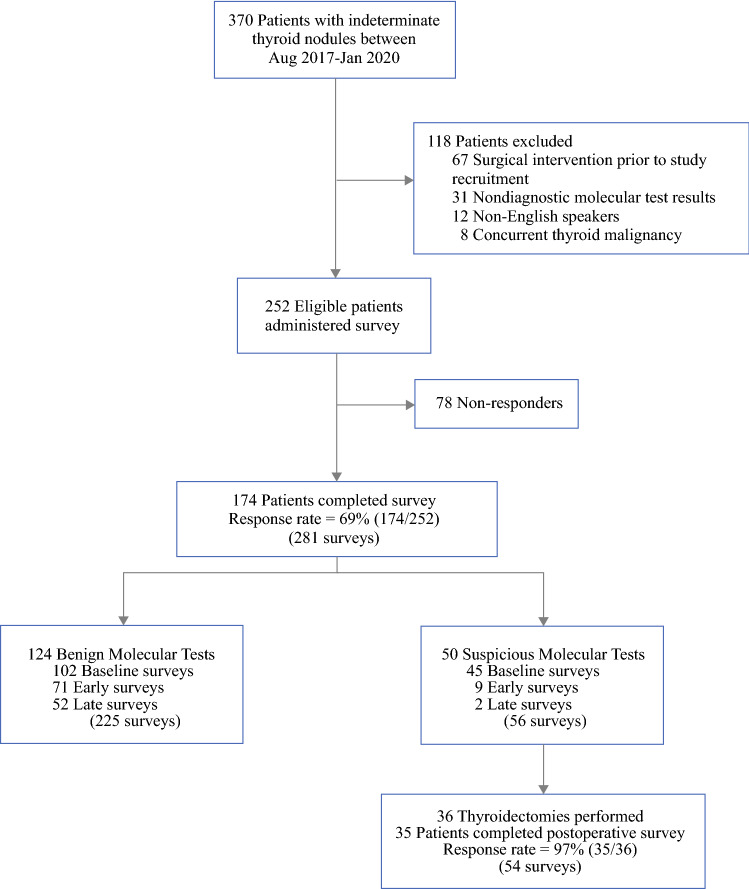
Flow diagram for all patients with indeterminate thyroid nodules who consented to complete the ThyPro-39 QOL study throughout UCLA Health (August 2017–January 2020). Baseline survey completed 0–4 months after FNA biopsy; early survey completed 4–12 months after FNA; late survey completed 12–24 months after FNA. *QOL* quality of life, *ThyPro-39* Thyroid-Related Patient-Reported Outcome, 39-item version, *UCLA* University of California Los Angeles, *FNA* fine needle aspiration

Of 370 patients with indeterminate thyroid nodules who underwent molecular testing from August 2017 to January 2020, 118 patients were excluded and 252 patients were eligible for the study; 174/252 patients completed the ThyPro-39 questionnaire (69% response rate)—124 (71%) with benign molecular tests and 50 (29%) with suspicious molecular tests (Fig. 1). Patients were excluded if they underwent surgical intervention prior to study recruitment (*n =* 67), were non-English speaking (*n =* 12), and had nondiagnostic molecular tests results (*n =* 31) or concurrent thyroid malignancy in another nodule (*n =* 8).

The median (interquartile range [IQR]) age of all participants was 56 years (44–66) and 77% were female (Table 1). The cytopathology results included 86.2% (*n* = 150) Bethesda 3 nodules and 13.8% (*n* = 24) Bethesda 4 nodules. Afirma GSC was performed in 54% of patients and Thyroseq v3 in 46%. Benign molecular test patients were less likely to have Bethesda 4 cytology than suspicious molecular test patients (9.7% vs. 24%, *p* = 0.03). No differences were observed in age, sex, nodule size, molecular test performed or TSH level between groups (Table 1)*.* The diagnostic performance of GSC and Thyroseq v3 has been published elsewhere.^14^

**Table 1:** Baseline demographic and clinical characteristics of survey respondents

	All cytologically indeterminate (*n* = 174)	Molecular test benign (*n* = 124) (69%)	Molecular test suspicious (*n* = 50) (31%)	*p*-value
Age, years				
Median (IQR)	56 (44–66)	58 (47–67)	53 (39–60)	0.20
<45 years	46 (26)	27 (21.3)	19 (38)	
Sex				1.00
Female	134 (77.0)	95 (76.6)	39 (78.0)	
Nodule size, cm				
Median (IQR)	2.0 (1.5–3.1)	2.0 (1.3–3.0)	2.4 (1.7–3.4)	0.06
<1	18 (10.3)	15 (12.1)	3 (6)	
1–2	69 (39.7)	51 (41.1)	18 (36)	
>2–4	76 (43.7)	53 (42.8)	23 (46)	
>4	11 (6.3)	5 (4.0)	6 (12)	
Bethesda category				0.03
AUS/FLUS	150 (86.2)	112 (90.3)	38 (76)	
SFN	24 (13.8)	12 (9.7)	12 (24)	
Molecular test				0.24
GSC	94 (54)	63 (50.8)	31 (62)	
ThyroSeq v3	80 (46)	61 (49.2)	19 (38)	
TSH, mU/L				0.24
Median (IQR)	1.7 (1.1–2.6) [*n =* 165]	1.7 (1.1–2.8) [*n =* 115]	1.6 (1.2–2.2)	
Hypothyroid, TSH >4.7	6 (3.6)	6 (5.2)	0 (0)	
Time to survey, months				
Baseline^a^ [median (IQR)]	1.9 (1.3–3.5)	2.0 (1.3–3.8) [*n =* 102]	1.7 (1.3–2.8) [*n =* 45]	0.23
Early^b^ [median (IQR)]	–	8.0 (7.3–9.6) [*n =* 71]	7.3 (4.9–7.7) [*n =* 9]	
Late^c^ [median (IQR)]	–	15.0 (13.8–18.9) [*n =* 52]	14.8 (13.8–15.7) [*n =* 2]	

Data are expressed as *n* (%) unless otherwise specified

*IQR* interquartile range, *AUS/FLUS* atypia of undetermined significance and follicular lesion of undetermined significance, *SFN* follicular neoplasm or suspicious for follicular neoplasm, *GSC* Afirma Genomic Sequencing Classifier, *TSH* thyroid-stimulating hormone, *FNA* fine needle aspiration

^a^Baseline, 0–4 months after FNA

^b^Early, 4–12 months after FNA

^c^Late, 12–24 months after FNA

Among the 124 patients with benign molecular test results, a baseline survey was completed in 102 patients at a median (IQR) of 2.0 months (1.3–3.8) after FNA, an early survey was completed in 71 patients at a median of 8.0 months (7.3–9.6) after FNA, and a late survey was completed in 52 patients at a median of 15.0 months (13.8–18.9) after FNA. Among the 50 patients with suspicious molecular test results, 45 completed a baseline survey at a median (IQR) of 1.7 months (1.3–2.8) after FNA. Most patients with suspicious molecular test results proceeded to thyroidectomy, thus follow-up surveys were included in the postoperative QOL analysis.

### Benign Versus Suspicious Molecular Test QOL

At baseline, patients with suspicious versus benign molecular tests reported worse symptoms of goiter, anxiety, and depression (Table 2). No QOL differences existed between groups at early follow-up.

**Table 2:** Quality-of-life comparisons at baseline, early and late follow-up among patients with indeterminate thyroid nodules

ThyPro-39 QOL scale	Baseline^a^	Early^b^	Late^c^
Goiter			
Molecular test benign	12.0 ± 15.0^d^ (*n* = 102)	11.4 ± 11.9 (*n* = 71)	11.3 ± 13.3 (*n* = 52)
Molecular test suspicious	21.1 ± 23.7 (*n* = 45)	11.1 ± 15.5^e^ (*n* = 9)	–
Anxiety			
Molecular test benign	24.8 ± 17.8^d^ (*n* = 102)	19.9 ± 22.1 (*n* = 71)	20.2 ± 22.8 (*n* = 52)
Molecular test suspicious	33.9 ± 26.6 (*n* = 45)	24.6 ± 19.1 (*n* = 9)	–
Depression			
Molecular test benign	24.0 ± 17.8^d^ (*n* = 102)	21.6 ± 15.0 (*n* = 71)	22.4 ± 17.3 (*n* = 52)
Molecular test suspicious	36.6 ± 22.6 (*n* = 45)	27.0 ± 15.1 (*n* = 9)	–
Emotional susceptibility			
Molecular test benign	26.3 ± 19.1 (*n* = 102)	24.3 ± 19.3 (*n* = 71)	25.9 ± 20.0 (*n* = 52)
Molecular test suspicious	31.8 ± 22.4 (*n* = 45)	33.0 ± 18.9 (*n* = 9)	–
Impaired social life			
Molecular test benign	14.5 ± 18.6 (*n* = 102)	13.1 ± 16.5 (*n* = 71)	11.6 ± 16.4 (*n* = 52)
Molecular test suspicious	19.2 ± 23.0 (*n* = 45)	11.1 ± 17.2 (*n* = 9)	–
Impaired daily life			
Molecular test benign	10.5 ± 16.4 (*n* = 102)	9.0 ± 14.8 (*n* = 71)	9.4 ± 14.1 (*n* = 52)
Molecular test suspicious	15.4 ± 21.0 (*n* = 45)	7.2 ± 9.2 (*n* = 9)	–
Appearance			
Molecular test benign	18.7 ± 23.0 (*n* = 102)	13.3 ± 19.6^e^ (*n* = 71)	14.3 ± 17.6 (*n* = 52)
Molecular test suspicious	18.5 ± 21.1 (*n* = 45)	10.9 ± 19.4 (*n* = 9)	–

QOL scale scores reported as mean ± standard deviation. Higher score corresponds to worse QOL. In ‘molecular test suspicious’ patients, early QOL assessment was only performed in those who did not undergo thyroidectomy

^a^Baseline, 0–4 months after FNA

^b^Early, 4–12 months after FNA

^c^Late, 12–24 months after FNA

^d^*p* < 0.05 compared with suspicious molecular test group at baseline

^e^*p* < 0.05 compared with baseline QOL score within the molecular test result group

*ThyPro-39* Thyroid-Related Patient-Reported Outcome, 39-item version, *QOL* quality of life

### Longtiudinal QOL

**Fig. 2.**
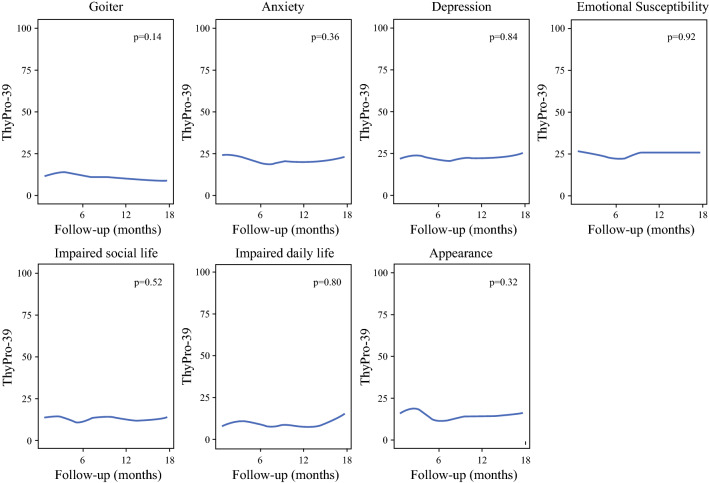
Change in ThyPro-39 QOL scale scores throughout ultrasound surveillance in benign molecular test result patients (*n* = 124). *ThyPro-39* Thyroid-Related Patient-Reported Outcome, 39-item version, *QOL* quality of life

On longitudinal assessment, no significant changes in symptoms of goiter, anxiety, depression, susceptibility, impact on social life, impact on daily life or appearance were observed in patients with benign molecular test results at early (relative to baseline) and late follow-up (Table 2). Appearance improved from baseline to early follow-up (mean ± standard deviation [SD] 18.7 ± 23.0 versus 13.2 ± 19.6, *p* = 0.048) but was this was not significant at late follow-up. As shown in Fig. 2, ThyPro-39 scores of benign molecular test patients assessed on linear regression showed no significant changes from time of baseline survey completion to 18 months of follow-up.

In patients with suspicious molecular test results managed nonoperatively with ultrasound surveillance, no differences were observed across all mean QOL scales at early follow-up, with the exception of improved symptoms of goiter (21.1 ± 23.7 [*n* = 45] vs. 11.1 ± 15.5 [*n* = 9], *p* = 0.04) (Table 2). No statistical tests were performed at late follow-up because of the small sample size at this time of assessment.

### Postoperative QOL for Suspicious Molecular Test

**Fig. 3.**
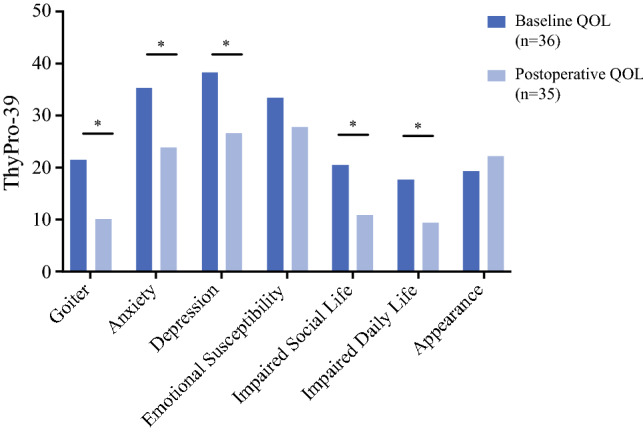
Comparison of baseline and postoperative ThyPro-39 QOL domains in patients with suspicious molecular test results who underwent thyroidectomy. Postoperative QOL assessment included 35 patients comprising 54 observations at a median of 8 months after thyroidectomy. * QOL significantly improved after thyroidectomy for symptoms of goiter, anxiety, depression, impaired social life, and impaired daily life (*p* < 0.05). *ThyPro-39* Thyroid-Related Patient-Reported Outcome, 39-item version, *QOL* quality of life

Among the 45 suspicious molecular test patients who completed baseline QOL assessment, 36 (80%) underwent thyroidectomy at a median (IQR) of 3.2 months (2.1–4.3) after FNA. Postoperative surveys were completed in 35 patients (97.2%) comprising a total of 54 observations. At a median (IQR) of 7.6 months (5.4–12.4) after thyroidectomy, suspicious molecular test patients reported improved symptoms of goiter (21.4 ± 23.9 vs. 9.9 ± 10.8, *p* < 0.01), anxiety (35.2 ± 28.0 vs. 23.9 ± 24.0, *p* = 0.049), depression (38.1 ± 23.5 vs. 26.6 ± 18.9, *p* < 0.01), impaired social life (20.4 ± 24.4 vs. 10.9 ± 18.1, *p* = 0.02), and impaired daily life (17.6 ± 22.5 vs. 9.4 ± 15.1, *p* = 0.04), relative to baseline (Fig. 3). In subgroup analyses, patients with benign surgical pathology (*n =* 23, 34 obervations) reported improved postoperative symptoms of goiter (21.4 ± 23.9 vs. 8.3 ± 9.6, *p* < 0.01) and impaired social life (20.4 ± 24.4 vs. 13.9 ± 20.7, *p* = 0.07, trend) (Table 3). In contrast, patients with malignant surgical pathology (*n =* 12, 20 observations) reported improvement in postoperative symptoms of depression (38.1 ± 23.5 vs. 20.4 ± 13.0, *p* < 0.01) and anxiety (35.2 ± 28.0 vs. 19.5 ± 22.4, *p* = 0.07, trend).

**Table 3:** Postoperative quality of life among patients with indeterminate thyroid nodules and suspicious molecular test results based on final surgical pathology

ThyPro-39 QOL scale	Baseline^a^ [*n* = 36]	Benign surgical pathology [*n* = 23]	Malignant surgical pathology [*n* = 12]
Goiter			
Mean ± SD	21.4 ± 23.9	8.3 ± 9.7*	12.5 ± 12.3
Anxiety			
Mean ± SD	35.2 ± 28.0	26.5 ± 24.9	19.5 ± 22.4
Depression			
Mean ± SD	38.1 ± 23.5	30.3 ± 21.0	20.4 ± 13.0*
Emotional susceptibility			
Mean ± SD	33.3 ± 22.2	29.9 ± 23.7	24.0 ± 20.6
Impaired social life			
Mean ± SD	20.4 ± 24.4	13.9 ± 20.7	5.9 ± 11.3
Impaired daily life			
Mean ± SD	17.6 ± 22.5	10.9 ± 16.6	6.8 ± 12.2
Appearance			
Mean ± SD	19.3 ± 21.8	27.6 ± 26.6	13.1 ± 20.6

Higher score corresponds to worse QOL. Postoperative surveys were completed at a median (interquartile range) of 8 months (5–12) after thyroidectomy

^a^Baseline = 0–4 months after FNA and prior to thyroidectomy

^b^*p* < 0.05 compared with baseline QOL

*ThyPro-39* Thyroid-Related Patient-Reported Outcome, 39-item version, *QOL* quality of life, *SD* standard deviation, *FNA* fine needle aspiration

Finally, we compared the postoperative QOL of patients with suspicious molecular tests and benign surgical pathology with those with benign molecular test results managed nonoperatively. We observed no significant differences in QOL when compared with benign molecular test patients at baseline. However, depression (30.3 ± 21.0 vs. 21.6 ± 15.0, *p* = 0.03) and appearance (27.6 + 26.6 vs. 13.3 ± 19.6, *p* = 0.01) were worse in suspicious molecular test patients with benign pathology versus benign molecular test patients at early assessment. Depression (30.3 ± 21.0 vs. 22.4 ± 17.3, *p* = 0.07, trend) and appearance (27.6 + 26.6 vs. 14.3 ± 17.6, *p* = 0.02) remained more severe when compared with benign molecular test patients at late follow-up.

## Discussion

Longitudinal assessment of QOL in patients with indeterminate thyroid nodules is an important measure to study as 90% of patients with benign molecular test results are managed nonoperatively,^18^ reducing the need for diagnostic thyroidectomy.^14^ While a benign molecular test result provides initial reassurance for patients with indeterminate thyroid nodules equivalent to benign cytopathology,^13^ QOL may be negatively impacted by residual concerns over the risk of malignancy and long-term clinical and sonographic surveillance. Our results indicate that a benign molecular test provides sustained QOL scores from the time of FNA through longitudinal surveillance, suggesting long-term reassurance of the low risk of malignancy. Patients with indeterminate nodules and suspicious molecular tests report worse QOL compared with those with benign molecular test results. Nevertheless, suspicious molecular test patients who underwent definitive surgery reported improvement in nearly all ThyPro-39 QOL domains, in relation to QOL at the time of molecular test diagnosis.

A benign molecular test resulted in sustained QOL from the time of FNA throughout follow-up and likely indicates the high level of physician and patient confidence in a benign molecular test diagnosis. In fact, appearance improved from baseline to early follow-up (18.7 vs. 13.2), which may be attributable to the overlap between a patient’s perception of appearance and their level of anxiety related to malignancy risk. For example, after initial diagnosis of an indeterminate nodule, a patient may potentially be more aware and concerned of the risk of malignancy, and with stable symptomatology and ultrasound findings over time, worry may diminish and reassurance gained. Although repeat FNA for Bethesda 3 indeterminate cytology may yield benign cytology in up to 42% of cases,^19^,^20^ which would likely also be reassuring, the remaining 58% of patients may continue to experience anxiety and fear regarding the possibility of thyroid cancer and the need for surgery. In the current study, a benign molecular test reduced unnecessary surgical resection^14^ while preserving health-related QOL.

Patients with suspicious versus benign molecular testing reported increased symptoms of goiter, anxiety, and depression following molecular test diagnosis. These findings confirm the results of a previous cross-sectional study assessing QOL in patients with indeterminate thyroid nodules, in which baseline ThyPro-39 scores for symptoms of goiter and depression were significantly worse in suspicious versus benign molecular test patients.^13^ In the current study, anxiety was also worse in suspicious molecular test patients (33.9 vs. 24.8). These differences in baseline QOL may be due to strong emotional reactions after a suspicious molecular test, causing worry regarding the possibility of cancer, the need to ‘get it out’^21^ and preparation for thyroidectomy, presenting as symptoms of anxiety, depression, and fear.^21^,^22^ A greater proportion of patients with suspicious molecular tests had nodule sizes >2 cm, which might explain why worse symptoms of goiter were reported by this group. In addition, the increased symptoms of depression and worry relating to the possibility of a cancer diagnosis may increase awareness of a globus sensation.^23^

Although the differences between benign and suspicious molecular test patients were no longer evident on early follow-up, this assessment is limited by the small sample size of suspicious molecular patients (*n =* 9) and self-selection bias, as these patients had deferred recommended surgical management and instead preferred observation. Interestingly, suspicious molecular test patients pursuing nonoperative management reported improved symptoms of goiter and a trend suggested improved anxiety and depression at 7 months of follow-up. Such patients who favor observation are likely more worried about the possibility of having surgery for their indeterminate thyroid nodule than the risk of malignancy it harbors. This improvement in QOL may therefore reflect satisfaction with their treatment decision. Physician interpretation of suspicious molecular test results and subsequent patient counseling and communication likely impacts QOL and represents one area that can be targeted to improve intial patient worry surrounding a possible cancer diagnosis.

Thyroid cancer patients experience symptom distress at the time of cytological diagnosis at a level comparable with general oncology patients, despite a generally good prognosis, and report disproportionately adverse physical, psychological, and social symptoms compared with patients with other malignancies.^9^,^24^,^25^ However, a paucity of data exist in those with indeterminate cytology. A recent qualitative study of 35 patients with cytologically indeterminate thyroid nodules demonstrated that despite worry about surgical scarring and postoperative complications, patients placed more importance on prompty removing the cancer due to fear of disease progression.^21^ These findings are supported in our study, as patients who underwent thyroidectomy for suspicious molecular test results demonstrated improved postoperative QOL in most ThyPro-39 domains, including anxiety and depression. Although these findings are limited by smaller sample sizes, we observed that indeterminate thyroid nodule patients with an ultimate cancer diagnosis on surgical pathology reported improvement in postoperative symptoms of depression (38.1 vs. 20.4) and anxiety (35.2 vs. 19.5), possibly related to confirming the diagnosis and having undergone treatment. In contrast, suspicious molecular test patients with benign surgical pathology reported worse depression and perceptions of appearance when compared with benign molecular test patients at early and late assessment. Patients with ultimately false positive molecular testing may feel they underwent an unnecessary operation and be adversely affected by a visible surgical scar. Our results provide an improved understanding of postoperative QOL specifically related to patients with indeterminate thyroid nodules and may guide pre- and postoperative counseling on active surveillance or immediate surgery in this setting.

Attention to QOL outcomes in thyroid cancer patients has been increasingly relevant given the indolent nature of most thyroid cancers. The negative QOL outcomes experienced by patients with indeterminate nodules at the time of suspicious molecular test diagnosis suggest that interventions should be considered to address this distress, given the favorable prognosis of most indeterminate thyroid nodules and thyroid cancers. Such measures could include improved patient counseling and education, social work consultation, and other forms of interdisciplinary care. Moreover, recent studies on molecular marker testing show improvements in diagnostic performance over prior versions.^26^,^27^ Increasing the benign call rate of future molecular testing iterations would further reduce unnecessary thyroidectomies while preserving the positive effects on QOL resulting from a benign molecular test.

Our study has several potential limitations. First, we were unable to evaluate the QOL effect of an indeterminate cytology result itself, as all indeterminate samples were reflexively sent for molecular testing. We felt that the inclusion of nondiagnostic molecular test patients was not valid as most of these patients underwent repeat molecular testing. Second, we were unable to assess QOL prior to FNA biopsy, which would evaluate the initial QOL of patients with a thyroid nodule and determine the subsequent impact of molecular test results on QOL. Third, we did not have specific data on patient’s race, ethnicity, socioeconomic status, or pre-existing conditions, including anxiety and depression, which may have been associated with baseline QOL. Third, we do not have surveillance data for benign molecular test patients who may have experienced nodule progression that could have impacted QOL. However, based on our previous results from ultrasound surveillance over a median follow-up of 27 months, 84% of nodules remained stable in size and appearance,^28^ therefore this variable is unlikely to significantly affect QOL outcomes observed in our study. Lastly, there was loss of patients during longititudinal follow-up, which may introduce nonresponse bias.

## Conclusion

We observed that benign molecular test patients reported similar QOL from the time of molecular test diagnosis through 18 months of follow-up. This finding suggests that a benign molecular test provides patients with sustained reassurance that the risk of malignancy is low. Definitive surgery resulted in improvement of QOL in patients with suspicious molecular test results.
